# Clinical genome editing to treat sickle cell disease—A brief update

**DOI:** 10.3389/fmed.2022.1065377

**Published:** 2023-01-09

**Authors:** Parinaz Zarghamian, Julia Klermund, Toni Cathomen

**Affiliations:** ^1^Institute for Transfusion Medicine and Gene Therapy, Medical Center — University of Freiburg, Freiburg, Germany; ^2^Center for Chronic Immunodeficiency (CCI), Faculty of Medicine, University of Freiburg, Freiburg, Germany; ^3^Ph.D. Program, Faculty of Biology, University of Freiburg, Freiburg, Germany

**Keywords:** base editing, clinical trial, CRISPR-Cas, γ-globin, gene editing, *HBB* gene, HbF

## Abstract

Sickle cell disease (SCD) is one of the most common hemoglobinopathies. Due to its high prevalence, with about 20 million affected individuals worldwide, the development of novel effective treatments is highly warranted. While transplantation of allogeneic hematopoietic stem cells (HSC) is the standard curative treatment approach, a variety of gene transfer and genome editing strategies have demonstrated their potential to provide a prospective cure for SCD patients. Several stratagems employing CRISPR-Cas nucleases or base editors aim at reactivation of γ-globin expression to replace the faulty β-globin chain. The fetal hemoglobin (HbF), consisting of two α-globin and two γ-globin chains, can compensate for defective adult hemoglobin (HbA) and reverse the sickling of hemoglobin-S (HbS). Both disruption of *cis*-regulatory elements that are involved in inhibiting γ-globin expression, such as BCL11A or LRF binding sites in the γ-globin gene promoters (*HBG1/2*), or the lineage-specific disruption of BCL11A to reduce its expression in human erythroblasts, have been demonstrated to reestablish HbF expression. Alternatively, the point mutation in the *HBB* gene has been corrected using homology-directed repair (HDR)-based methodologies. In general, genome editing has shown promising results not only in preclinical animal models but also in clinical trials, both in terms of efficacy and safety. This review provides a brief update on the recent clinical advances in the genome editing space to offer cure for SCD patients, discusses open questions with regard to off-target effects induced by the employed genome editors, and gives an outlook of forthcoming developments.

## Introduction

Sickle cell disease (SCD) is one of the most common hemoglobinopathies, which comprises a group of disorders that are characterized by faulty hemoglobin production (1, 2). Hemoglobin, a two-way respiratory carrier in red blood cells (RBCs), is responsible for transporting oxygen to tissues and returning carbon dioxide to the lung. This tetrameric metalloprotein is composed of two α-subunits, two non-α-subunits, hem groups, and four iron atoms, giving hemoglobin the capacity for binding oxygen (3). For congenital forms of anemia, SCD and thalassemia have the highest incidence (4). According to the European Medicines Agency (EMA) and the U.S. Center for Disease Control and Prevention (CDC), approximately 20 million people worldwide, including 52,000 people in Europe and 100,000 Americans, are affected by SCD. These patients suffer from anemia as well as progressive and fatal cardiovascular, renal, and eye complications due to the abnormal sickling shape of the RBCs that causes clogging of capillaries (1, 2). To alleviate morbidity, current treatment options include regular blood transfusions and the application of drugs that prevent vaso-occlusive crisis (VOC) or that reduce erythrocyte sickling. Still, life expectancy is reduced due to progressive organ dysfunction (1, 2). The only approved curative option for SCD is allogeneic hematopoietic stem cell (HSC) transplantation, which requires the availability of “healthy” blood stem cells of siblings or non-related donors with matched human leukocyte antigen (HLA). Unfortunately, the difficulty of finding suitable donors early in childhood and the high risk of graft-vs.-host-disease limit the option of bone marrow transplantation for SCD patients (5, 6). One way to overcome this limitation is the use of autologous HSCs that are corrected *ex vivo* using gene therapy strategies to restore functional hemoglobin expression. Because of its genetics, SCD represents an ideal target for gene therapy in general and for genome editing in particular.

## Hemoglobin expression

**FIGURE 1:**
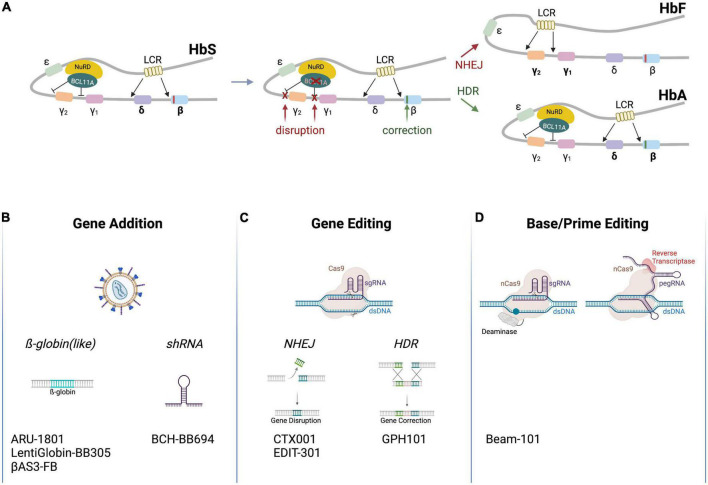
Schematic of clinical genome editing approaches for SCD. **(A)** The β-globin locus. The locus encompasses *HBE* (encoding ε-globin), *HBG2* and *HBG1* (γ-globin), *HBD* (δ-globin) and *HBB* (β-globin). A locus control region (LCR) and various factors (depicted are BCL11A and NuRD) regulate developmental stage-specific expression of the hemoglobin genes. A point mutation in *HBB* (red box) leads to expression of HbS. Therapeutic gene editing strategies aim at correcting *HBB via* HDR (green box) or at disrupting *cis*-regulatory elements in the *HBG2/HBG1* promoters or in *BCL11A via* NHEJ to re-activate γ-globin expression (red Xs), resulting in the expression of HbA (α_2_β_2_) or HbF (α_2_γ_2_), respectively. **(B–D)** Platform technologies used for the treatment of SCD. Clinically employed are **(A)** lentiviral vectors to transfer β-globin like genes or an shRNA targeting BCL11A mRNA, **(B)** genome editors to disrupt *cis*-regulatory elements by NHEJ or correct *HBB* by HDR, or **(C)** base and prime editors to disrupt *cis*-regulatory elements or off-set the mutation in *HBB*. The respective autologous, genome-engineered cell products are listed on the bottom (Created with BioRender.com).

The two non-α-subunits of hemoglobin are encoded by five different genes located within the β-globin locus on chromosome 11 (Figure 1A). The respective genes, *HBE* (coding for ε-globin), *HBG2* and *HBG1* (γ-globin), *HBD* (δ-globin) and *HBB* (β-globin), are expressed in a developmental stage-specific manner in erythroid cells (7). A single locus control region (LCR) and specific enhancers are responsible for their sequential activation during development. In the early stage embryonic yolk sac, *HBE* is expressed. Later, hematopoiesis shifts to the liver and the *HBG1/HBG2* genes (which are the result of a gene duplication and produce proteins that only differ in one amino acid) are activated to produce fetal hemoglobin (HbF, α_2_γ_2_). Shortly after birth, hematopoiesis relocates to the bone marrow, and *HBD* and *HBB* are expressed, leading to an almost complete replacement of HbF by adult hemoglobin HbA (>95% α_2_β_2_, 1.5–3.5% α_2_δ_2_; with 0.6–1% HbF persisting) (8). The γ-globin to β-globin switch is mediated by different transcription factors that repress *HBG1/HBG2* expression, such as BCL11A and LRF (9, 10). Worthy of note, healthy individuals with a benign genetic condition called hereditary persistence of fetal hemoglobin (HPFH) exhibit persistent production of functional HbF even after birth. The molecular basis of HPFH are large deletions in the *HBD* and *HBB* genes, which increase interactions between the LCR and the *HBG1/HBG2* promoters (11), or alternatively mutations in the *cis*-regulatory elements of the *HBG* genes, which are bound by the transcriptional repressors BCL11A and LRF (9, 10). If these repressors can no longer bind to the said *cis*-regulatory elements, *HBG* expression—and hence HbF production—persists (12).

Sickle cell disease arises as a result of a homozygous mutation in the *HBB* gene, in which a single point mutation leads to a codon change from gAg to gTg, resulting in a valine to glutamic acid substitution on the protein level (2). This swap in position six affects the hydrophobic characteristics of hemoglobin, converting HbA into the so-called sickle hemoglobin (HbS, α_2_β*^S^*_2_)—a term deduced from the sickle-like shape of the RBCs upon polymerization of HbS into fibers under deoxygenated conditions. The kinetic of hemoglobin polymerization is sensitive to the concentration of the HbS. Of note in this context, SCD patients with HPFH mutations present with mild clinical manifestations because *HBG* reactivation enables the formation of α_2_γ_2_ and α_2_γβ*^S^* on top of α_2_β*^S^*_2_. Furthermore, the glutamine at position 87 (Q87) of γ-globin was shown to inhibit HbS polymerization and increase HbS solubility under deoxygenated conditions, so adding to the anti-sickling activity.

## Gene therapy for SCD

The earliest attempts to genetically treat SCD were based on lentiviral (LV) transfer of a functional *HBB* copy to autologous HSCs (13). Bluebird Bio initiated first phase I/II gene therapy clinical trials in 2013 in France with seven patients (4 transfusion-dependent β-thalassemia, TDT, 3 SCD; HGB-205, NCT02151526) and in 2014 in the U.S. with 50 SCD patients (HGB-206, NCT02140554). The clinical product, LentiGlobin BB305 (Figure 1B), entails autologous HSCs transduced with an LV that encodes an anti-sickling variant of β-globin, known as βA-T87Q (mimicking the inhibitory effect of HbF on HbS polymerization). The recently published results confirmed stable βA-T87Q expression upon engraftment as well as reduced hemolysis, absence of VOC, and transfusion-independency (13, 14). A phase III clinical study (NCT04293185) with 35 SCD patients as well as a long-term follow-up study (NCT04628585) were opened in 2020. Based on these pivotal studies (15, 16), BB305 received marketing authorization from the EMA (17) and the FDA (18) under the trade name Zynteglo^®^ for the treatment of transfusion-dependent β-thalassemia (TDT). Of note, two patients from the phase I/II BB305 study (NCT02140554) were diagnosed with acute myeloid leukemia (AML) 2 years post-infusion (19), but AML development was not linked to insertional mutagenesis. The chosen conditioning regimen and/or the proliferative stress on HSCs upon switching from homeostatic to regenerative hematopoiesis might have played a role in AML induction and/or progression (14, 19).

Because high HbF expression ameliorates symptoms associated with SCD (20), efforts to develop LV-based approaches to increase γ-globin expression have been undertaken. This includes an LV expressing a γ-globin^*G*16*D*^ variant that was shown to have increased affinity to α-globin (21). Clinical data (NCT02186418) showed long-lasting engraftment with potentially curative HbF levels (21). The Boston Children’s Hospital initiated a phase I clinical study with 10 patients in 2018 (NCT03282656) using autologous HSCs that were transduced with an LV (BCH-BB694) encoding a short-hairpin micro-RNA (shmiR) targeting the BCL11A mRNA (22). The six patients with long-term follow-up (7–29 months) showed high levels of HbF, mild clinical disease manifestation and no SAEs, prompting a phase II trial (NCT05353647) with 25 participants in 2022. Despite these successes, the high manufacturing costs of LV vectors (23), their potential of instigating abnormally spliced transcripts (24), as well as the risk of genotoxicity due to semi-random integration (25), limit the application of LV-based therapies.

## Genome editing to treat SCD

Genome editing enables the site-specific modification of the human genome in order to correct or offset mutations underlying genetic disorders (26). Genome modification typically ensues from DNA double strand breaks (DSBs) that are introduced by programmable designer nucleases, such as zinc finger nucleases (ZFNs) (27), transcription activator-like effector (TALE) nucleases (TALENs) (28, 29), or the CRISPR-Cas system (30). Other than the entirely protein-based ZFNs and TALENs, CRISPR-Cas nucleases contain an engineered guide (gRNA) that is complementary to the desired target sequence and that directs the Cas protein to the chosen genomic locus to induce a DSB (Figure 1C). Non-homologous end joining (NHEJ) and HDR are the two major repair pathways triggered by DSB formation (31). NHEJ is a fast but error-prone pathway, leading to insertions and deletions at the break site. NHEJ is hence typically employed to disrupt genes or *cis*-regulatory elements with high efficacy, reaching editing frequencies of over 90% in HSCs (26). In contrast, HDR is a slow but precise DNA repair pathway that uses a co-introduced DNA fragment as a template to correct disease underlying mutations *inter alia*. In HSCs, the HDR template is typically delivered by vectors based on adeno-associated virus (AAV) (32) or in the form of single-stranded or double-stranded oligonucleotides (ODNs) (33). However, because HDR is restricted to the S/G2 phase of the cell cycle, achieving gene targeting frequencies that exceed 20% in mainly quiescent long-term repopulating HSCs remains challenging (34).

Due to the genotoxic potential arising from DSB formation (see below), alternative platforms to edit the genome have been sought for Figure 1D. Such strategies are mostly based on CRISPR-Cas nickases that cleave only one DNA strand (35–37). This family includes base editors (BEs) (38, 39) and prime editors (PEs) (40). A Cas9 nuclease is converted to a Cas9 nickase by introducing mutations in one of the two catalytic domains of Cas9 (36). BEs were developed by fusing a deaminase domain to a Cas nickase (38). There are two types of BEs: cytosine base editors (CBEs) convert a C•G base pair (bp) into a T•A while adenine base editors (ABEs) convert an A•T to a G•C bp. BEs can be employed to correct point mutations, to introduce stop codons, or to disrupt *cis*-regulatory elements. PEs consist of a Cas9 nickase coupled to an engineered reverse transcriptase, which transcribes a section of the pegRNA (prime editing gRNA) into DNA to introduce the desired changes, such as base conversions or insertions/deletions of up to 80 bp (40).

## Genome editing clinical trials for SCD

In the last 4 years, seven clinical trials using gene editing technologies to treat SCD have been initiated (Table 1). In all of them the editing agents are delivered *ex vivo* to autologous HSCs. Five of these therapeutic approaches attempt to reactivate γ-globin expression, either by preventing *BCL11A* expression in the erythroid lineage through disruption of enhancer elements or by mutating the BCL11A binding sites in the *HBG* promoters (Figure 1A). Two alternative approaches aim to correct the disease-causing mutation in the *HBB* locus using HDR (Figure 1A).

**TABLE 1:** Gene editing clinical trials for sickle cell disease.

Clinical trial	Phase	Year started	Treatment name	Target gene	Delivery mode	Designer nuclease	Donor template	Sponsors	Location	Status
NCT05329649	III	2022	CTX001	*BCL11A*	RNP electroporation	CRISPR-Cas9	–	Vertex Pharmaceuticals, CRISPR Therapeutics	United States, Italy	Recruiting
NCT05477563	III	2022	CTX001	*BCL11A*	RNP electroporation	CRISPR-Cas9	–	Vertex Pharmaceuticals, CRISPR Therapeutics	United States	Recruiting
NCT04774536	I/II	2022	CRISPR-SCD001	*HBB*	RNP electroporation	CRISPR-Cas9	ssODN	University of California	United States	Not yet recruiting
NCT05456880	I/II	2022	BEAM-101	*HBG1/HBG2*	RNA electroporation	ABE base editor	–	Beam Therapeutics	United States	Not yet recruiting
NCT05145062 (long-term follow up)	N/A	2021	BIVV003	*BCL11A*	mRNA electroporation	Zinc finger nuclease	–	Sangamo Therapeutics	United States	Recruiting
NCT04208529 (long-term follow up)	N/A	2021	CTX001	*BCL11A*	RNP electroporation	CRISPR-Cas9	–	Vertex Pharmaceuticals, CRISPR Therapeutics	United States, Canada, Germany, Italy, UK	Enrolling by invitation
NCT04819841	I/II	2021	GPH101	*HBB*	RNP electroporation	CRISPR-Cas9	rAAV6	Graphite Bio	United States	Recruiting
NCT04853576	I/II	2021	EDIT-301	*HBG1/HBG2*	RNP electroporation	CRISPR-Cas12a	–	Editas Medicine	United States, Canada	Recruiting
NCT04443907	I/II	2020	OTQ923	*BCL11A*	Unknown	CRISPR-Cas9	–	Novartis Pharmaceuticals, Intellia Therapeutics	United States, Italy	Recruiting
NCT03653247	I/II	2019	BIVV003	*BCL11A*	mRNA electroporation	Zinc finger nuclease	–	Sangamo Therapeutics	United States	Recruiting
NCT03745287	II/III	2018	CTX001	*BCL11A*	RNP electroporation	CRISPR-Cas9	–	Vertex Pharmaceuticals, CRISPR Therapeutics	United States, UK, Canada, France, Italy, Belgium, Germany	Active, not recruiting

The most advanced product, CTX001, was developed by CRISPR Therapeutics and Vertex Pharmaceuticals. It is currently being tested in CLIMB-121, a phase II/III clinical trial (NCT03745287) that was started in 2018 with 45 SCD patients. CTX001 is administered as an autologous HSC product edited with CRISPR-Cas9 to disrupt the lineage-specific enhancer in the *BCL11A* gene. This alteration reduces BCL11A expression in erythroid cells, which in turn reactivates γ-globin expression. Published clinical data from the first two patients (one SCD and one TDT patient) demonstrated a high level of edited alleles in the stem cell compartment (69% and 80%). At 15 months post-transplantation, HbF levels in the SCD patient rose from 9.1 to 43.2%, while HbS levels were reduced from 74.1 to 52.3%. Patients were reported to be transfusion-independent and free of VOC. A recent update from infusion of CTX001 in 44 TDT and 31 SCD patients confirmed the overall positive response: All patients presented a sustained increase in HbF (39.6–49.6%), improvement in mean total Hb level (>11 g/dl) after 3 months, as well as elimination of VOC. Bone marrow analyses (>12 months follow-up) confirmed durable effects of this therapy over time with > 80% edited alleles. On the other hand, several severe adverse events (SAEs) were observed in patients upon infusion of the edited cells, such as VOC liver disease, sepsis, cholelithiasis, and hemophagocytic lymphohistiocytosis (HLH). Non-serious lymphopenia was also reported, most likely due to a delay in lymphocyte recovery (41, 42).

In 2019, Sangamo Therapeutics started a phase I/II clinical trial (NCT03653247) for eight SCD patients to assess the safety and efficacy of BIVV003, *ex vivo* manufactured autologous HSCs that were edited with ZFN technology to disrupt the *BCL11A* erythroid-specific enhancer. Data from week 26 post-transplantation of four patients showed increased HbF levels (14–39%) and F-cells raised to 48–94%. VOC was reported in one patient with a low level of HbF (14%). BIVV003 was well tolerated without the need for transfusions post-transplantation in all four patients (43). Besides adverse events related to plerixafor-based mobilization of CD34^+^ cells and busulfan conditioning, no SAEs related to the treatment were reported (43).

Conversely, it was reported that editing of the *BCL11A* erythroid enhancer can result in reduced erythroid output, which was not observed when the binding site of BCL11A in the *HBG* promoters was disrupted (44). Editas Medicine initiated in 2021 the phase I/II RUBY clinical trial (NCT04853576) with almost 40 participants to evaluate the efficacy and safety of EDIT-301, a product based on autologous HSCs in which the *HBG1*/*2* promoter regions are disrupted using CRISPR-Cas12a. In preclinical mouse models, long-term engraftment of *HBG1/2*-edited HSCs was observed. The ∼90% edited target alleles went along with a high-level of HbF induction in cells from healthy donors (43%) and SCD patients (54%) with no detectable off-target effects (16, 44).

The 2021 initiated CEDAR trial (NCT04819841) is a phase I/II clinical study sponsored by Graphite Bio. As opposed to the previously described products, GPH101 is based on HDR and relies on a high-fidelity CRISPR-Cas9 system in combination with an AAV6-based HDR template. The goal is to correct the SCD-underlying point mutation in *HBB*. In preclinical mouse studies, almost 20% of HSCs harbored a corrected *HBB* locus (32), resulting in 90% of RBCs with normal HbA. The preclinical safety data revealed no evidence of abnormal hematopoiesis as well as absence of detectable off-target activity or chromosomal translocations. Graphite Bio recently announced the enrollment of the first patient, with up to 15 patients following at multiple sites in the U.S. Initial data from the CEDAR trial are expected for mid 2023.

Beam Therapeutics started a phase I/II clinical trial with 15 enrolled SCD patients in 2022 (NCT05456880). In BEAM-101, γ-globin expression is activated through a base swap in the *HBG1/2* promoters using base editing to generate an HPFH genotype variant in autologous HSCs. Based on preclinical mouse data, > 90% of target sites in xenotransplanted HSCs were stably edited, resulting in high levels of γ-globin expression (>65% HbF) (45). Furthermore, an investigational new drug application was filed for BEAM-102, which was designed to change the point mutation in *HBB* from gTg to gCg. The result is a switch from glutamic acid to alanine in position 6, which converts HbS into a better tolerated HbG-Makassar (46).

Technical challenges of *ex vivo* genome editing approaches in HSCs are similar to those in LV-based approaches and comprise to reach a sufficient number of mobilized CD34 + cells as a starting material, sufficient editing efficacy in the LT-HSC compartment, a lower level of engraftment of *ex vivo* edited cells along with reduced stemness of edited HSCs (47–49).

## Off-target effects

Similar to insertional mutagenesis associated with integrating vector systems, inadvertent on-target and off-target effects evoked by the genome editing tools represent a major concern when applied in patient cells, particularly in highly proliferating multipotent stem cells. On the one hand, cleavage by CRISPR-Cas nucleases can trigger undesired effects on the target chromosome (50, 51), such as large deletions and inversions (52–54), chromosomal truncations (55), chromothripsis (56), aneuploidy (57), loss of heterozygosity (58), and loss of imprinting (58). On the other hand, unintentional activity at so-called off-target sites, that is sequences with high homology to the intended target site, triggers NHEJ-mediated insertion/deletion mutations at off-target sites and, potentially, comparable structural aberrations as described for the on-target site. Moreover, concomitant insertions of DSBs at multiple sites in the genome elicit translocations between those cleaved sites (54, 59). Several methods to predict or detect off-target activity and/or gross chromosomal rearrangements have been developed. They include deep sequencing of *in silico* predicted off-target sites as well as experimental procedures that detect off-target activity *in vitro* and in cell-based systems. Commonly used *in vitro* methods include CIRCLE-Seq (60), ONE-Seq (61) and NucleaSeq (62), while GUIDE-Seq (63), DISCOVER-Seq (64) and CAST-Seq (54) are prevalently used cell-based approaches. Noteworthy, CAST-Seq not only nominates off-target sites but also detects chromosomal rearrangements at the on-target site as well as induced chromosomal translocations with off-target sites (54).

The gene-edited products that are currently employed in clinical trials typically underwent several genotoxicity tests as part of the preclinical risk assessment. For instance, off-target activities of the CRISPR-Cas nucleases used in CTX001 and GPH101 were profiled by GUIDE-Seq, CIRCLE-Seq, and targeted amplicon next-generation sequencing (Amp-Seq) of *in silico* predicted off-target sites. Similarly, the safety of EDIT301 was investigated with GUIDE-Seq and Amp-Seq of *in silico* predicted off-target sites. Given that translocations are a hallmark of leukemic cells (65, 66) and since they can be rather frequent outcomes of genome editing (54, 59, 67, 68), there is a growing interest in detecting gross structural rearrangements, such as large chromosomal deletions, inversions, truncations, and translocations, too. To our knowledge, many of the above-mentioned products did not undergo a genome-wide and sensitive analysis of induced chromosomal rearrangements. Against the backdrop of the high sequence similarities within the β-globin locus (*HBG1* vs. *HBG2* or *HBB* vs. *HBD*), the potential for off-target editing as well as homology-mediated recombination between two respective paralogous genes is high (69). Indeed, rearrangements between *HBB* and *HBD* were confirmed in *HBB*-edited cell, in addition to translocations between *HBB* and an off-target site (54, 70, 71). In addition, CRISPR-Cas nucleases targeting either *HBB*, *HBD* or *HBG1/HBG2* can lead to complete loss of the distal chromosome 11p arm in HSCs (58). Furthermore, the simultaneous disruption of the BCL11 binding sites in *HBG1* and *HBG2* was reported to result in deletion of the 4.9 kb region between the two target sites, eliminating *HBG2* in 5–30% of cells (72–74).

To avoid this loss of the *HBG2* gene, BEs were employed to introduce HPFH-like mutations in the *HBG1/HBG2* promoters (75). Because single-strand nicks are repaired by the high-fidelity base-excision repair pathway, BEs have been claimed to reduce on-target and off-target effects (36). However, recent data demonstrated deletion of a 4.9 kb region after base editing of the *HBG1/HBG2* promoters, indicating that also base editing can induce structural variations (76). Furthermore, bystander editing effects (77) and gRNA-independent off-target activities on both DNA and RNA (78, 79) have been described for both ABEs and CBEs. Hence, additional efforts are needed to characterize BE-associated off-target effects as well as to identify gross chromosomal rearrangements triggered by editing tools in HSCs of SCD (and TDT) patients. This also includes the evaluation of the biological long-term effects of genotoxicity in transplanted patients as well as the development of strategies to mitigate the observed off-target effects.

## Future developments for SCD-directed genome editing

Genotoxic conditioning regimens still pose a major barrier to the adoption of autologous HSC transplantation in SCD (80, 81). To overcome this problem, Beam Therapeutic, among others, is developing a new approach termed “engineered stem cell antibody paired evasion,” in which a BE-introduced epitope switch in CD117 enables those CD117-edited HSCs to selectively escape CD117-directed antibody-based conditioning. Such a strategy can be easily applied to BEAM-101 by targeting *CD117* and the *HBG1/2* promoters simultaneously (82).

Are there additional transcription factors that could be targeted to upregulate γ-globin expression? MYB is a transcription factor that regulates fetal hemoglobin expression at multiple levels, including upregulation of BCL11A expression (83). ATF4 is further upstream and regulates the expression of MYB. It has been recently shown that knockout of *ATF4* lowered MYB—and hence BCL11A—expression, and could thus potentially re-activate γ-globin expression (84). However, it must be noted that MYB and ATF4 have multiple functions outside of HbF regulation in non-erythroid cells (85, 86), highlighting the need to identify erythroid-specific regulation.

Given the constraints of off-target effects associated with all genome editing platforms, the question is whether alternatives to genome editing are available. Several studies deciphered the epigenetic regulation of the β-globin locus during development, including the interaction between epigenetic and transcriptional regulation leading to repression of γ-globin expression (87, 88). This knowledge opened up the idea to modify the epigenome in a targeted fashion for the treatment of SCD. While epigenetic approaches to promote γ-globin re-expression were described before (89, 90), more specific approaches are needed for clinical translation. Designer epigenome modifiers based on the TALE or CRISPR-dCas9 platforms create an opportunity to manipulate the epigenetic marks specifically and without the necessity to induce breaks in the genome (91, 92), e.g., by rewriting the epigenetic code in order to re-activate *HBG* expression or to silence *BCL11A* in a lineage-specific manner. Epigenome modifiers might therefore have less deleterious effects in a cell. On the other hand, the challenge of maintaining long-lasting effects over several cell cycles and throughout lineage differentiation has not been solved yet and it will be interesting to see whether the potential of designer epigenome modifiers can be harnessed for the treatment of SCD in the near future (93, 94).

## Author contributions

All authors contributed to the writing and proofreading of the manuscript.
